# Assessment of intrafractional prostate motion and its dosimetric impact in MRI-guided online adaptive radiotherapy with gating

**DOI:** 10.1007/s00066-022-02005-1

**Published:** 2022-09-23

**Authors:** Yuqing Xiong, Moritz Rabe, Lukas Nierer, Maria Kawula, Stefanie Corradini, Claus Belka, Marco Riboldi, Guillaume Landry, Christopher Kurz

**Affiliations:** 1 Department of Radiation Oncology, University Hospital, LMU Munich, Munich, Germany; 2 Partner Site Munich, German Cancer Consortium (DKTK), Munich, Germany; 3 Department of Medical Physics, Ludwig-Maximilians-Universität München (LMU Munich), Garching (Munich), Germany

**Keywords:** MR linac, Prostate cancer, Organ motion, MRI-guided radiotherapy, Dose reconstruction

## Abstract

**Purpose:**

This study aimed to evaluate the intrafractional prostate motion captured during gated magnetic resonance imaging (MRI)-guided online adaptive radiotherapy for prostate cancer and analyze its impact on the delivered dose as well as the effect of gating.

**Methods:**

Sagittal 2D cine-MRI scans were acquired at 4 Hz during treatment at a ViewRay MRIdian (ViewRay Inc., Oakwood Village, OH, USA) MR linac. Prostate shifts in anterior–posterior (AP) and superior–inferior (SI) directions were extracted separately. Using the static dose cloud approximation, the planned fractional dose was shifted according to the 2D gated motion (residual motion in gating window) to estimate the delivered dose by superimposing and averaging the shifted dose volumes. The dose of a hypothetical non-gated delivery was reconstructed similarly using the non-gated motion. For the clinical target volume (CTV), rectum, and bladder, dose–volume histogram parameters of the planned and reconstructed doses were compared.

**Results:**

In total, 174 fractions (15.7 h of cine-MRI) from 10 patients were evaluated. The average (±1 σ) non-gated prostate motion was 0.6 ± 1.0 mm in the AP and 0.0 ± 0.6 mm in the SI direction with respect to the centroid position of the gating boundary. 95% of the shifts were within [−3.5, 2.7] mm in the AP and [−2.9, 3.2] mm in the SI direction. For the gated treatment and averaged over all fractions, CTV D_98%_ decreased by less than 2% for all patients. The rectum and the bladder D_2%_ increased by less than 3% and 0.5%, respectively. Doses reconstructed for gated and non-gated delivery were similar for most fractions.

**Conclusion:**

A pipeline for extraction of prostate motion during gated MRI-guided radiotherapy based on 2D cine-MRI was implemented. The 2D motion data enabled an approximate estimation of the delivered dose. For the majority of fractions, the benefit of gating was negligible, and clinical dosimetric constraints were met, indicating safety of the currently adopted gated MRI-guided treatment workflow.

## Introduction

Prostate cancer was the most common cancer among the male population of Germany in 2020, with an incidence rate of 23.5% and almost 68,000 new diagnoses [1]. For localized prostate cancer, patients most commonly undergo radical prostatectomy or whole prostate radiotherapy [2]. Within the scope of external beam radiation therapy, state-of-the-art delivery techniques such as intensity-modulated radiation therapy (IMRT) and volumetric modulated arc therapy (VMAT) enable tight dose conformation. Hence, accurate prostate positioning becomes crucial [3, 4]. Today, cone-beam computed tomography (CBCT) and implanted fiducial markers are often used for patient setup on each treatment day. With these, the prostate can be aligned with respect to the planned treatment position via in-room imaging to account for interfractional variation of the prostate position [5, 6]. This approach assumes that the prostate remains static during the treatment fraction and disregards intrafractional motion of the prostate caused by internal organ motion, such as changes in rectal and bladder filling. Huang et al. and Mah et al. found that the prostate can move up to 1.3 cm in the anterior–posterior (AP), 1.1 cm in the superior–inferior (SI), and 1.0 cm in the left–right (LR) direction during a treatment fraction [7, 8]. The main concern with intrafractional prostate motion is loss of target coverage. For instance, Nejad-Davarani et al. found a decrease of up to 20.2% in the dose received by 95% of the planning target volume (PTV) due to prostate displacement during a period of about 45 min [9].

Ideally, both inter- and intrafractional motion needs to be taken into consideration when establishing a prostate treatment protocol. In clinical practice, a PTV margin around the clinical target volume (CTV) is added to account for prostate motion and patient setup errors during treatment planning [10, 11]. However, this margin should be kept as small as reasonably possible so that the volume of organs at risk (OARs) in the high-dose region is minimized [2].

In the past decade, the integration of a magnetic resonance imaging (MRI) scanner and a linear accelerator (linac), called an MR linac, was introduced in clinics. There are currently two commercially available MR linac systems [12], the MRIdian by ViewRay (ViewRay Inc., Oakwood Village, OH, USA; first patient on the cobalt-60 system in 2014 and on MR linac in 2017) [13–15] and the Unity by Elekta (Elekta AB, Stockhom, Sweden, first patient in 2017) [16, 17]. MRI is considered to be advantageous over CBCT for image guidance in radiotherapy due to the dose-free imaging capabilities and improved soft tissue contrast [18]. Based on the enhanced soft tissue contrast, the accuracy of patient positioning and anatomical structure delineations are improved. In addition, the treatment plan can be reoptimized at each treatment fraction with the help of in-room MR images and corresponding delineations, following an MRI-guided online adaptive radiotherapy workflow [19, 20]. Real-time cine MRI data can be acquired during patient irradiation and used to gate the beam delivery, where the treatment beam is only switched on when the target is located within a predefined boundary [15, 21]. For prostate cancer treatment, initial studies have shown that the use of MRI-guided radiotherapy (MRgRT) can achieve equivalent treatment results to conventional radiotherapy, and that late toxicities are low [22–24]. Initial studies to understand how the prostate intrafractional motion can have an impact on the delivered dose have been conducted at the Elekta Unity, which does not offer gated beam delivery at the time of writing. Kontaxis et al. [25] and Menten et al. [26] have both reported a slight underdosage of the CTV for non-gated treatments.

Schaule et al. found that the MRI-guided online adaptation remained dosimetrically beneficial compared to no adaptation within a timespan of 45 min [27] in a study with healthy volunteers. Over longer timespans of 60 min, however, considerable degradation of the target coverage caused by intrafractional target motion was observed. When taking the in-room preparation time and treatment delivery time into account, this time threshold can be exceeded in clinical practice. Although it is possible to mitigate the dose degradation caused by prostate intrafractional motion via gating at the ViewRay MRIdian, residual motion within the predefined gating boundary is still present, and its dosimetric impact remains unclear and will be investigated in this study. Furthermore, the effect of gating itself is of interest. This study aimed at creating a pipeline to extract the prostate motion captured during MRI-guided online adaptive radiotherapy using 2D cine MRI data. The pipeline extracts the motion during beam-on time, which represents the residual motion within the gating window, as well as the full, non-gated motion. The delivered dose was reconstructed by exploiting the dose cloud approximation [28, 29] and the dosimetric impact of motion with and without gating was examined.

## Materials and methods

### Patient cohort

Ten prostate cancer patients were included in this study. All patients underwent MRI-guided online adaptive radiotherapy with gated beam delivery at a 0.35 T MR linac (MRIdian, ViewRay Inc., Oakwood Village, OH, USA) [15] at the Department of Radiation Oncology at the University Hospital of LMU Munich. Two patients underwent hypofractionated treatment with 5 × 7.25 Gy, one patient was treated with 30 × 2.00 Gy, and the remaining seven patients received 20 fractions of 3.00 Gy. More details on the patients are provided in Table 1. The duty cycle was defined as the ratio of beam-on time to the delivery time with image pauses. The duration of cine videos did not necessarily match with the delivery time because the cine MR imaging was paused at every gantry rotation. A gating event was defined as the transition between the beam being on and switched off when the target status changed from in to out.

**Table 1:** Patient information: age, TNM (tumor, lymph node, metastases) stage, Gleason score, dose prescription, number of collected cine MRI videos, CTV size, average delivery time with and without pauses caused by gantry rotations, duty cycle, total number of repositionings, and gating events over all fractions

Patient	Age [years]	TNM stage	Gleason score	Prescription [Gy]	Collected fractions	CTV size [cm^3^]	Mean delivery time (without pauses) [min]	Mean delivery time (with pauses) [min]	Duty cycle [%]	Total number of repositionings	Total number of gating events
1	74	T2a, N0, Mx	7	5 × 7.25	5	38.8	10.0	13.2	41.4	1	4
2	70	T2a, N0, M0	6	20 × 3.00	20	69.8	4.5	7.4	34.7	1	8
3	77	T3a, N0, M0	6	30 × 2.00	30	37.4	4.6	7.8	23.5	1	10
4	74	T2b, N0, M0	7	20 × 3.00	20	60.6	5.8	9.0	29.1	0	0
5	74	Tx, Nx, M1	9	5 × 7.25	5	50.8	7.4	10.4	58.6	3	9
6	75	T3a, Nx, Mx	6	20 × 3.00	20	104.5	6.1	9.2	27.3	4	26
7	75	T2c, N0, Mx	7	20 × 3.00	20	51.3	4.6	7.5	38.8	0	0
8	80	T2b, N0, M0	9	20 × 3.00	19	59.3	7.9	11.0	27.6	1	36
9	65	T2c, N0, Mx	7	20 × 3.00	20	52.6	3.7	6.6	39.2	1	37
10	81	T2b, N0, M0	6	20 × 3.00	15	59.9	4.7	7.4	31.7	6	15

The number of collected videos does not always match the prescribed number of fractions due to delivery of a few fractions at conventional linacs. The MR linac was under maintenance due to technical problems on these days

*CTV* clinical target volume

### Clinical workflow

In the planning stage, a planning MRI (pMRI) and a planning CT (pCT, Aquilion, Canon Medical Systems; voxel size: 1.0 × 1.0 × 3.0 mm^3^) of the patient in the supine treatment position were acquired. The pMRI was acquired at the MR linac (balanced steady-state free precession sequence; bSSFP) with an in-plane resolution of 1.5 × 1.5 mm^2^ and a slice thickness of either 1.5 mm or 3.0 mm (TR/TE: 3.4 ms/1.4 ms; acquisition matrix: 334 × 300 × 288 or 334 × 300 × 144; in-plane field-of-view, FOV: 501 × 450 mm^2^; flip angle: 60°. TR: repetition time, TE: echo time). Contour delineation was performed on the pMRI. The pCT was deformably registered to the pMRI to provide electron density information for treatment planning. All patients were treated with step-and-shoot IMRT plans (9 to 21 beams) which were created in the ViewRay MRIdian treatment planning system (version 5.2.5.14).

The PTV enclosed the CTV with an isotropic margin of 4.5 mm, except posteriorly towards the rectum. Here, to decrease the dose towards the rectum, an expansion of 3.0 mm of the CTV was used [28]. Planning aimed to cover at least 95% of the PTV with the prescribed dose.

All patients were instructed to follow a drinking and eating protocol for defined bladder and rectum filling on the days of treatment (see Appendix). At each fraction, the same 3D bSSFP MRI sequence as used for pMRI was acquired and used for contour delineation and possibly plan adaption. A new synthetic CT (sCT) was generated by pMRI-to-daily-MRI deformable image registration. OAR segmentations were transferred to the daily MRI by the same deformable registration, while target structures were transferred via rigid registration. All segmentations were adjusted manually by an experienced radiation oncologist and the baseline treatment plan was recalculated on the sCT, to decide whether or not to adapt the baseline plan [29].

For beam gating during dose delivery, a 2D sagittal cine MRI was acquired using a bSSFP sequence with a framerate of 4 Hz and an in-plane resolution of 3.5 × 3.5 mm^2^ (slice thickness: 5 mm; TR/TE: 2.4/1.1 ms; acquisition matrix: 100 × 100 or 78 × 78; FOV: 350 × 350 mm^2^ or 270 × 270 mm^2^; flip angle: 60°). To gate the dose delivery, an additional boundary contour was defined on the corresponding sagittal MRI slice, which was obtained by isotropically expanding the CTV with a margin in the [3.0, 5.0] mm range. The CTV was chosen as tracking contour. During treatment, the boundary contour stayed static, while the target contour was deformed by the vendor’s optical flow algorithm to match the moving anatomy.

**Fig. 1:**
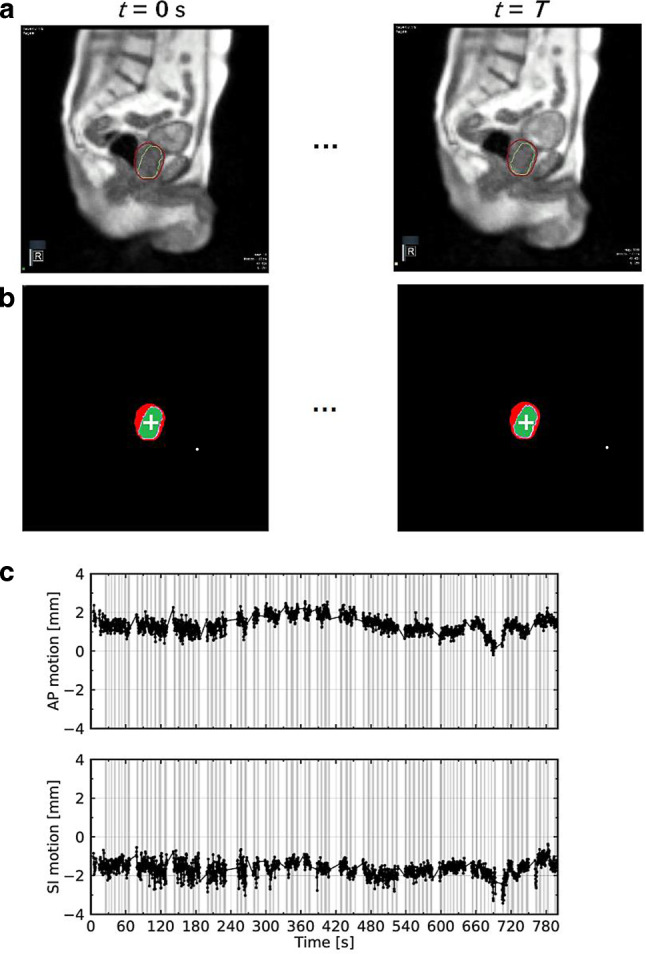
Extraction of the centroid motion of the target contour in anterior-posterior and superior–inferior directions from the cine MRI data for a typical fraction (patient 1, fraction 3). **a** Cine MRI frames at the beginning (*t* = 0 s) and the end (*t* = *T*) of the fraction. Target (*green*) and boundary (*red*) contours are overlaid on the MR image. The coloured square in the left lower corner shows the beam status (*green*: beam off, *yellow*: beam on). **b** Contour filling and extraction of the centroid position of the target (*white cross*) and the boundary. **c** Obtained motion curve of the moving target centroid relative to the static boundary centroid (*grey shaded area*: beam-on phases). Interruptions in the curves are due to imaging pauses during gantry rotation

The photon beam was switched off if 5% of the target’s area was located outside of the boundary. The fraction was interrupted by the operator when the target position was outside for too long, thus hindering beam delivery. In these cases, the patient was repositioned and irradiation was resumed. A screenshot of a typical frame from a patient’s cine MRI video is shown in Fig. 1a. In total, 174 cine MRI videos with a cumulative duration of 15.7 h were collected for this study (Table 1).

### Motion extraction

The 2D target motion over treatment time *t* in the AP and SI directions is captured in the (2D + *t*) cine MRI data. To extract the motion trajectories and the beam status during each fraction, in-house software developed in Python (version 3.8.3) was implemented. With RGB value thresholds, both target and boundary contours were extracted from the video frames. These two contours were then filled homogenously using the watershed algorithm [30]. From these filled contours, the centroid positions of the boundary and the target contours were computed. Finally, the time-resolved relative shifts between the centroid position of the target and the fixed boundary centroid position were calculated. The main steps of the procedure are depicted in Fig. 1. Firstly, the full, non-gated motion was extracted frame-by-frame from the entire cine MRI video for each fraction. Target shifts along AP and SI directions were extracted separately. Secondly, according to the beam status displayed on each frame, the beam-on shifts were selected for further analysis. These corresponded to the residual motion within the gating window, which we labeled gated motion.

The mean values of the non-gated and gated motion in AP and SI directions over all fractions and patients were computed, together with the corresponding standard deviations (σ) as well as the range of 95% of the motion amplitudes.

### Dose reconstruction

For each treatment fraction, the 3D fractional dose distribution and the corresponding delineations were exported from the ViewRay treatment planning system. The 3D fractional dose originated from either the original non-adapted plan (in cases where the treatment was not adapted) or the adapted plan (153/174 fractions were adapted). For interrupted fractions, the dose distributions from sub-fractions were merged, together with the prostate motion extracted from the separate cine MRI data series. All sub-fractions shared the same segmentation.

Since it was not possible to have access to the linac or multileaf collimator log files, a time-resolved segment-by-segment dose reconstruction could not be implemented. As an approximation for reconstructing the delivered dose, the static dose cloud approach was used [31, 32]. The static 3D fractional dose distribution was shifted rigidly with the inverse of the gated 2D prostate motion vectors extracted from the 2D cine MRI. The shifted dose distributions were averaged to estimate the delivered fractional dose, labelled gated dose. To reconstruct the dose of a hypothetical non-gated delivery, the same approach was adopted using the non-gated motion.

For a comparison of these reconstructed doses with the static planned fractional dose, the clinically relevant dose–volume-histogram (DVH) parameters D_98%_ of the CTV and D_2%_ of the rectum and the bladder were calculated. The relative differences between reconstructed and planned dose CTV D_98%_ were calculated δ_D98%_ = (ΔD_98%_/D_98%_) × 100% using the CTV D_98%_ of the static planned fraction dose. The same was done to define δ_D2%_ = (ΔD_2%_/D_2%_) × 100% for the OARs.

## Results

### Motion analysis

**Fig. 2:**
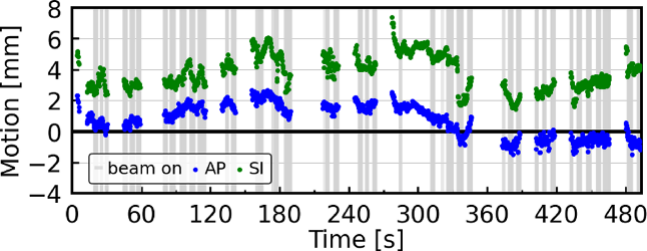
Intrafractional motion in anterior–posterior (*blue*) and superior–inferior (*green*) directions for an example fraction of patient 7. The *grey bands* indicate frames with the beam switched on. Interruptions in the curves are due to pauses for gantry rotation. The gating boundary margin was 5 mm for this patient

The non-gated motion curves of a fraction with relatively large prostate displacements are shown in Fig. 2 (mean AP shift: 0.8 mm, 95% range: [−1.1, 2.4] mm; mean SI shift: 3.8 mm, 95% range: [1.8, 5.8] mm). Averaged over all patients and fractions, the mean ±1 σ shifts in the AP and SI directions for non-gated motion were −0.6 ± 0.9 mm and 0.0 ± 0.6 mm, respectively. For gated motion, the mean ±1 σ shifts were −0.6 ± 0.8 mm in the AP and 0.0 ± 0.6 mm in the SI direction.

For non-gated motion, 95% of the target displacements were between [−3.5, 2.7] mm in the AP and between [−2.9, 3.2] mm in the SI direction. For gated motion, 95% of the displacements were between [−3.3, 2.7] mm and [−2.9, 3.0] mm in the AP and SI directions, respectively.

**Fig. 3:**
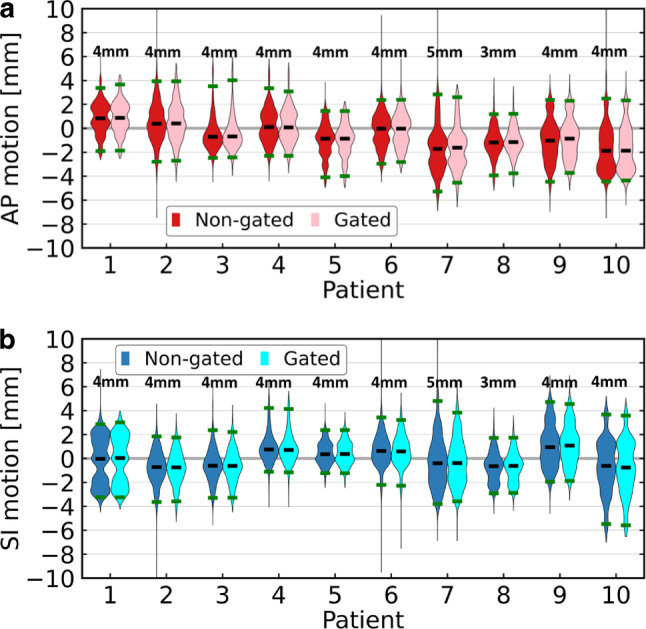
Violin plots of the target displacement in anterior–posterior (*AP*; **a**) and superior–inferior (*SI*; **b**) directions over all fractions for each patient. Gated motion is plotted side-by-side with the non-gated motion. Shifts beyond 10 mm are not shown. In each violin plot, the *black horizontal line* shows the median. 95% of the data points are located between the *two green horizontal lines*. The *upper and lower ends* of a violin plot mark the two extrema. The size of the isotropic gating window of each patient is indicated above the violin plots. Shifts beyond 10 mm are not displayed for improved visibility

Violin plots of prostate motion over all fractions for each patient are shown in Fig. 3. The non-gated motion and the gated motion are plotted side-by-side. The median target displacement in SI and AP over all fractions was between −2.0 mm and 1.0 mm for both gated and non-gated motion. For nine out of the ten patients, the gated motion amplitude was less than 6.0 mm in the AP and SI directions. Per patient, the 95% intervals of non-gated motion in the AP and SI directions were within [−5.3, 3.9] mm and [−5.5, 4.8] mm, respectively. For gated motion, 95% intervals were within [−4.5, 4.0] mm in the AP and [−5.6, 4.6] mm in the SI direction.

### Dosimetric analysis

**Fig. 4:**
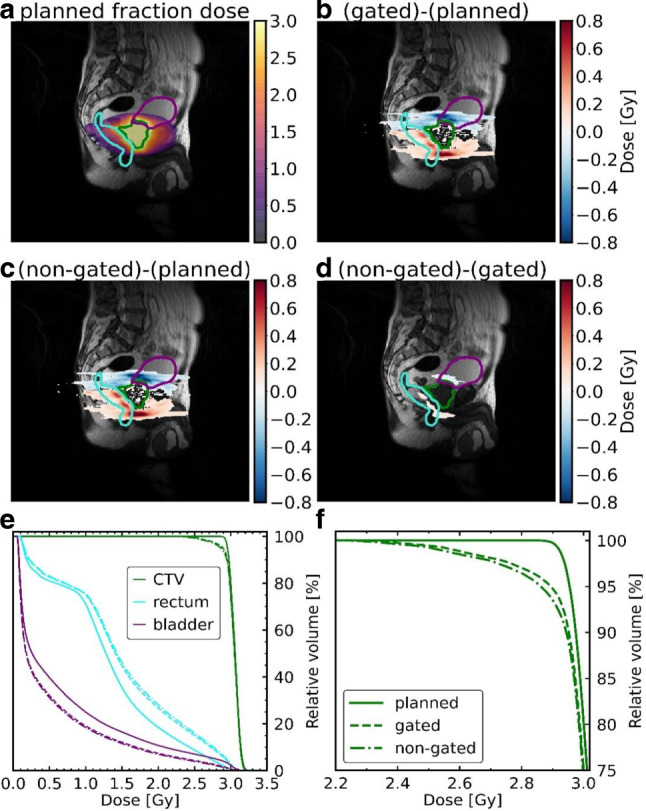
Dose comparison of an exemplary fraction with strong motion of patient 7 (fraction dose: 3.00 Gy). The planned static dose (**a**) is compared to the reconstructed gated (**b**) and non-gated (**c**) doses. Differences of the latter two are shown in **d** (dose differences lower than 0.05 Gy are not displayed) with clinical target volume (*CTV*; *green*), rectum (*cyan*), and bladder (*purple*). **e** shows the corresponding dose–volume histogram of the fraction, with a zoomed-in view in **f**. *Solid curve*: planned fraction dose; *dashed curve*: reconstructed gated fraction dose. *Dashed-dotted curve*: non-gated fraction dose

Fig. 4 shows the dose differences corresponding to the example fraction shown in Fig. 2. On average, the prostate was shifted towards the superior–anterior direction. As a result, the CTV and the bladder received a lower dose, while the dose in the rectum increased, as can be seen in the DVH in Fig. 4. For this fraction, the gated CTV D_98%_ decreased by 0.22 Gy (non-gated: decrease by 0.26 Gy), with δ_D98%_ = 7.5% (non-gated δ_D98%_ = 8.9%). The gated rectum D_2%_ increased by 0.12 Gy (non-gated: 0.12 Gy), with δ_D2%_ = 4.0% (non-gated δ_D2%_ = 4.1%), while the gated bladder D_2%_ decreased by 0.16 Gy (non-gated: 0.20 Gy), with δ_D2%_ = 5.2% (non-gated δ_D2%_ = 6.7%). The same evaluation was performed for every fraction of every patient.

**Fig. 5:**
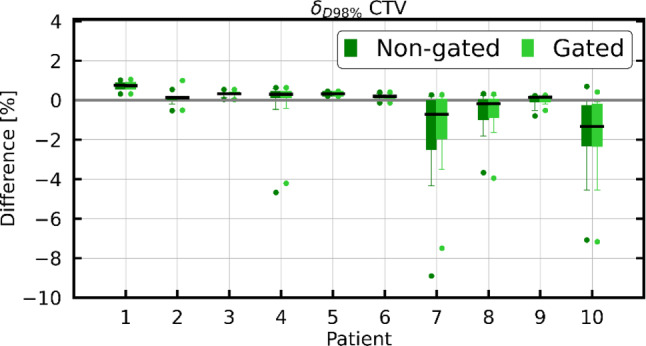
Boxplots of CTV δ_D98%_ (non-gated in *dark green*, gated in *light green*). The *black line* in each boxplot marks the median, while the *upper and lower whiskers* encompass 95% of the data. *CTV *clinical target volume

The CTV δ_D98%_ of all fractions of each patient are shown as boxplots in Fig. 5. As mentioned, for each fraction, the gated dose and non-gated dose were separately compared with the planned static dose. The maximum difference for gated delivery was −7.5% (−0.22 Gy) and −8.9% (−0.26 Gy) for non-gated delivery at individual fraction level. Over all fractions of each individual patient, the maximum median difference was −1.4% (−0.04 Gy) for both gated and non-gated delivery. The mean ±1 σ of the CTV δ_D98%_ over all fractions and patients was −0.2 ± 0.8% (−0.01 ± 0.02 Gy) and was the same for both gated and non-gated scenarios. While for some patients δ_D98%_ was close to 0% over all fractions, for other patients, larger deviations in the CTV D_98%_ were observed during fractions with larger mean prostate displacements. Differences between gated and non-gated doses were mainly observed for these fractions (see for example patient 7 in Fig. 4).

**Fig. 6:**
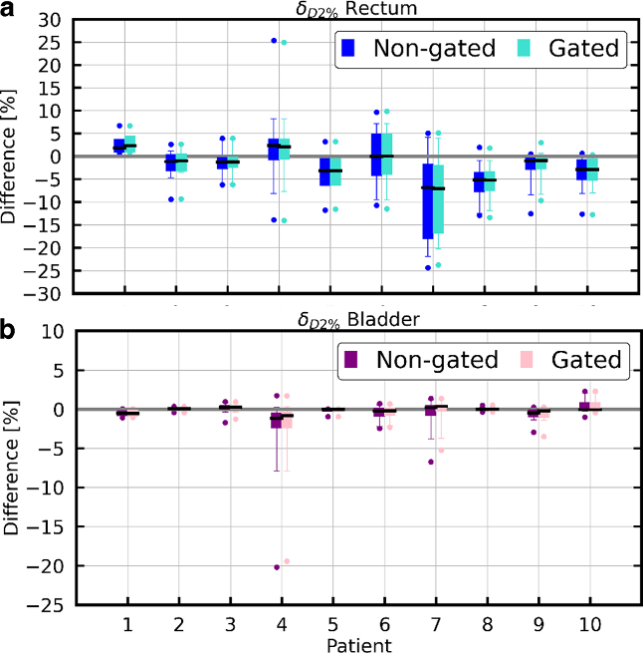
Boxplots of δ_D2%_ for the rectum (**a**) and bladder (**b**; non-gated in *dark color* and gated in *light color*). The *black line* in each boxplot marks the median, while the *upper and lower whiskers* encompass 95% of the data

Similarly, the OARs’ δ_D2%_ for all fractions of each patient are depicted in Fig. 6. An increase in rectum D_2%_ by up to 25% (0.59 Gy) of the planned D_2%_ was found in single fractions. The deviations between planned and reconstructed dose in the bladder were smaller. The absolute differences between non-gated and gated δ_D2%_ were smaller than 2.9% for rectum and 2.2% for bladder.

The bladder D_2%_ increased by less than 5% of the planned D_2%_ in all gated and non-gated fractions. Averaged over all fractions (±1 σ), δ_D2%_ was −2.3 ± 3.3% (−0.07 ± 0.13 Gy) for rectum and −0.4 ± 0.7% (−0.01 ± 0.02 Gy) for bladder for both gated and non-gated delivery.

## Discussion

During an average cine MRI, the average (±1 σ) non-gated target shift was −0.6 ± 1.0 mm in the AP and 0.0 ± 0.6 mm in the SI direction. Over all patients, the average 95% interval of prostate displacements in the AP and SI directions for non-gated motion were [−3.5, 2.7] mm and [−2.9, 4.2] mm, respectively. The average 95% intervals in both directions were within the chosen PTV–CTV margin of 4.5 mm in every direction except the posterior direction, where a reduced margin of 3.0 mm was applied. In combination with the selected gating window (CTV with an isotropic expansion in the [3.0, 5.0] mm range), an efficient beam usage without too many interruptions due to the target moving out of the gating boundary could be achieved, even though the motion amplitude could vary considerably from fraction to fraction, and between patients. According to our findings (see Fig. 3), the gating window was able to filter out large motions and furthermore enabled intervention by the treatment team when target displacements exceeded the boundary for a certain time.

These results agree with the range of motion reported by Keizer et al. (mean AP shift: 1.1 mm; mean SI shift: −1.2 mm) [33] and Menten et al. (mean AP shift: 0.6 mm; mean SI shift: −1.3 mm) [26]. With the help of 3D cine MRI, Keizer et al. showed that the prostate motion in the LR direction was negligible compared to motions in the other two directions (3D cine MRI temporal resolution of 0.12 Hz). Based on this, Menten et al. extracted the prostate motion only from 2D cine MRI (temporal resolution of 1.63 Hz) in sagittal orientation. These studies indicate that prostate motion analysis using 2D cine MR images was sufficient for the intended purpose of this study. Due to challenges in target contour registration, prostate rotation was not included in this study. In contrast to Keizer et al. and Menten et al., patients included in our study underwent gated delivery at a ViewRay MR linac. If the prostate drifted outside of the gating window for an extended period, the treatment was interrupted and the patient was repositioned. Therefore, important prostate motions exceeding the extent of the gating window were compensated for during beam delivery and the shifts determined in our study are smaller than the motion without interruptions and repositioning. A correlation between the average treatment time per fraction and the size of the target shifts was not observed.

The full non-gated motion extracted from the entire cine MRI was used to reconstruct the dose for a non-gated delivery, while the beam-on motion was applied for reconstructing the gated delivery dose. Averaged (±1 σ) over all patients, the CTV D_98%_ of the gated fraction dose decreased by 0.2 ± 0.8%. Averaged over all treatment fractions, the CTV D_98%_ decreased by less than 2.0% for all individual patients for both gated and non-gated delivery. These results imply that the intrafractional residual motion within the gating window led to a small underdosage in the CTV, but sufficient target coverage was still achieved for all cases. For rectum and bladder, the D_2%_ increased by up to 25% and 3%, respectively, in single gated and non-gated fractions. Averaged over all fractions, however, for every patient, the rectum and the bladder D_2%_ increase was below 3.1% and 0.5%, respectively. Therefore, the current clinical choice of the CTV–PTV margin and gating boundary can be deemed safe. The rectum was found to be more sensitive to motion-induced variations than the bladder. This is most likely attributed to its elongated shape, smaller volume, and proximity to the prostate. Nevertheless, DVH parameters of the rectum and bladder fulfilled the clinical constraints for the majority of fractions. Improvement of CTV coverage and OAR protection by a gated delivery in comparison to non-gated delivery was only observable for single fractions during which the target had larger motion amplitudes. In our study, the impact of gating was relatively small. However, larger motions observed on the cine-MRI triggered interruption of the treatment fraction in several cases (see Table 1), which was only possible with the help of target tracking and beam gating. The extent of the benefit of delivery interruptions could not be estimated because the information on the non-gated motion without repositioning of the patient, i.e., in a scenario where the fraction would not have been interrupted, was not available. Potentially, the gating boundary could thus be enlarged further to improve the treatment efficiency by reducing the number of dose delivery interruptions. Such conclusions depend, however, on the choices of the CTV–PTV margin as well as the size of the gating boundary.

Kontaxis et al. and Menten et al. also observed underdosage of the CTV (Kontaxis et al. D_99%_ average change of −2.2 ± 2.9%; Menten et al. CTV D_98%_ average change of −1.8 ± 2.7%). Additionally, Menten et al. also observed variations of 0.0 ± 2.0% and −1.7 ± 3.3% in the rectum and bladder D_3%_. The changes in the CTV reported by these two studies are more pronounced than in our study. These differences are likely caused by an interplay of various factors. Firstly, since these studies did not include gating, larger motions were likely and had an impact on the delivered dose distribution. Secondly, for our study, the dose cloud approximation was chosen due to the lack of access to machine log files. Consequently, a segment-by-segment dose reconstruction considering the corresponding prostate displacement, as proposed by Kontaxis et al. and Menten et al., was not feasible. As a result, our reconstructed dose might not be an optimal representation of the actual dose delivery with modulated beam intensities and potential interplay effects of target motion and dynamic beam delivery. Also, the static dose cloud approximation itself might not perfectly estimate the shifted dose distribution, but was found sufficient to reconstruct the dose in adaptive prostate radiation therapy, as shown by Sharma et al. and Unkelbach et al. [34, 35]. In particular, the relevant effects due to drift motion are likely captured correctly.

A continuous volumetric 3D model of patient anatomy containing rotations and deformations would be necessary to provide a more sophisticated anatomic representation of the prostate for future studies, in combination with access to the machine log files. For dose accumulation using such a dynamically updating anatomic model, fast time-resolved 3D MRI and, especially when the deformations are strong, a reliable deformable image registration method is required [36, 37]. This would also allow to include information of the (less pronounced) left–right motion, which was neglected in our approach. Over the past few years, different approaches to infer time-resolved 3D anatomy from orthogonal 2D cine MRI have been demonstrated [38–40] with promising results. The accuracy of this study could also be further improved if access to the raw DICOM data of the 2D cine MR images and the tracking structures was facilitated, so that extraction of the target motion from the resampled postprocessed cine MRI videos would not be required.

The tools developed within the scope of this study might in the future be applied to further anatomic sites such as lung and pancreas, which additionally suffer from pronounced respiratory-induced motion and potentially enhanced residual motion within the defined gating window. However, due to the heterogeneity of the tissue, the static dose cloud approach may not be sufficiently accurate for dose reconstruction. In the future, for better generalizability of the conclusions of this study, the patient cohort might be extended and more data included.

In conclusion, a workflow was developed in this study to extract the intrafractional prostate motion captured during online adaptive gated MRgRT based on 2D cine MRI and to reconstruct the delivered dose using a dose cloud approximation. The obtained results of motion and dosimetric analyses demonstrate that the current clinical PTV margins and gating window sizes for prostate cancer MRgRT at our department are adequate and safe. The algorithms might in the future also support estimation of the treatment efficiency and quality in different margin or gating window scenarios to further optimize MRgRT for prostate cancer.

## Appendix

### Drinking and eating protocol

All patients were advised to follow the protocol below for both planning CT/MRI and subsequent irradiation sessions:

- The patient should visit the toilet for the last time one hour before the beginning of the treatment fraction.
- Afterwards, the patient is recommended to drink about 0.75 L of water.
- The patient should try to empty his/her rectum about 2–3 h before the appointment for irradiation.
- The patient is recommended to have multiple smaller meals during the day. Vegetables and nuts which can cause flatulence in the abdominal area should be avoided. Furthermore, strong spices and sparkling drinks should be off the menu.
